# Increased Activity‐Dependent Bulk Endocytosis in Huntington's Disease Results From Huntingtin Haploinsufficiency

**DOI:** 10.1111/jnc.70134

**Published:** 2025-06-21

**Authors:** Han C. G. Tan, Robyn L. McAdam, Andrew Morton, Michael A. Cousin, Karen J. Smillie

**Affiliations:** ^1^ Centre for Discovery Brain Sciences, Hugh Robson Building University of Edinburgh Edinburgh Scotland UK; ^2^ Muir Maxwell Epilepsy Centre, Hugh Robson Building University of Edinburgh Edinburgh Scotland UK; ^3^ Simons Initiative for the Developing Brain, Hugh Robson Building University of Edinburgh Edinburgh Scotland UK

**Keywords:** endocytosis, haploinsufficiency, Huntington's disease, mouse, presynapse, synaptic vesicle

## Abstract

Huntington's disease (HD) is a life‐limiting, progressive monogenic neurodegenerative disorder characterised by chorea, hypokinesis and psychosocial symptoms. HD is characterised by a variable CAG expansion in exon 1 of the *HTT* gene, which encodes the huntingtin (htt) protein. This expansion results in an extended polyglutamine tract, which is widely thought to confer a toxic gain of function on the protein that is responsible for disease progression. Most individuals with HD are heterozygous for this mutation, meaning that loss of wild‐type htt function may also contribute to disease pathology. We previously identified that the recycling of synaptic vesicle proteins at the presynapse was specifically disrupted in striatal neurons from a preclinical model of HD, the *Htt* ^Q140/Q140^ knockin mouse. This defect was only revealed during high activity and, notably, was due to loss of wild‐type htt function. The dominant endocytosis mode at the presynapse during high activity is activity‐dependent bulk endocytosis (ADBE). Therefore, we determined whether dysfunction in this pathway was linked to this recycling defect. We revealed that three independent neuronal subtypes derived from *Htt* ^Q140/Q140^ mice displayed enhanced recruitment, but no change in the extent of ADBE via the evoked uptake of fluid phase markers. Importantly, this phenotype was due to a loss of wild‐type htt function, since depletion of htt in *Htt* ^+/+^ neurons mimicked the defect, and removal of mutant htt from *Htt* ^Q140/Q140^ neurons did not correct this dysfunction. Neurons from *Htt* ^Q140/+^ mice, which mimic the human condition, also displayed increased activity‐dependent triggering of ADBE, suggesting that htt haploinsufficiency may be responsible. This was confirmed by the inability of zinc finger proteins that selectively target mutant htt to correct this defect in *Htt* ^Q140/+^ neurons. Therefore, htt haploinsufficiency drives dysfunction in a key endocytosis mode that is dominant during high neuronal activity, providing a potential mechanism for circuit dysfunction that results in neurodegeneration in later life in HD.

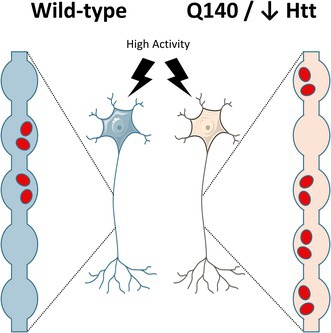

## Graphical abstract

Huntington's Disease (HD) is a neurodegenerative disorder characterised by an expanded CAG repeat mutation in the *HTT* gene, causing an extended polyglutamine tract in the huntingtin (htt) protein. We reveal that different neuronal subtypes derived from mice that model HD (Q140) display enhanced recruitment of activity‐dependent bulk endocytosis, the dominant presynaptic endocytosis mode during high activity. This increase results from loss of wild‐type htt function, not a toxic gain of function. Therefore, htt haploinsufficiency drives dysfunction in an endocytosis mode dominant during high neuronal activity, providing a potential mechanism for circuit dysfunction that contributes to neurodegeneration in HD.

**Abbreviations**
AAV: adeno‐associated virus
ADBE: activity‐dependent bulk endocytosis
AP5: 2‐amino‐5‐phosphonopentanoic acid
BDNF: brain‐derived neurotrophic factor
BSA: bovine serum albumin
CAG: cytosine alanine guanine triplicate
CGN: cerebellar granule neurons
CME: clathrin‐mediated endocytosis
CNQX: 6‐cyano‐7‐nitroquinoxaline‐23‐dione
DIV: days in vitro
DMEM‐F12: Dulbecco's modified eagle medium: nutrient mixture F‐12
E: embryonic day
GFP: green fluorescent protein
GSK3: glycogen synthase kinase 3
GTPase: guanosine triphosphatase
HD: Huntington's disease
HEPES: 4‐(2‐hydroxyethyl)piperazine‐1‐ethanesulfonic acid
HPC: hippocampal neurons
HRP: Horseradish peroxidase
hsiRNA: hydrophobically modified small interfering RNA
htt: huntingtin protein
mhtt: mutant huntingtin protein
MSNs: medium spiny neurons
NTC: non‐targeting control
P: post‐natal day
PBS: phosphate‐buffered saline
PFA: paraformaldehyde
Q: glutamine
ROI: region of interest
STR: striatal neurons
SV2A: synaptic vesicle protein 2A
SVs: synaptic vesicles
syp‐pH: synaptophysin‐phluorin
TMR‐dextran: tetramethylrhodamine‐dextran
ZFP: zinc finger protein

## 1 Introduction

Huntington's disease (HD) is a monogenic disorder characterised by a variable CAG expansion in exon 1 of the *HTT* gene, resulting in the expressed huntingtin protein (htt) containing an extended polyglutamine tract. The disease results from a specific degeneration of medium spiny neurons (MSNs) in the striatum and is characterised by chorea followed by hypokinesis (Vonsattel et al. 1985). The emergence of symptoms and progression of the disease, which typically manifests in middle age, are strongly associated with the length of the expanded polyglutamine tract (Saudou and Humbert 2016). Brain development and function are also dysregulated in early life in a number of HD model systems (Humbert 2010), suggesting htt performs key roles throughout the lifespan of an individual.

An emerging theme in a number of neurodevelopmental and neurodegenerative conditions is the premise that presynaptic dysfunction plays a causal or contributory role towards later pathological outcomes (Waites and Garner 2011; Bonnycastle et al. 2021). The efficient and accurate recycling of synaptic vesicles (SVs) at the presynapse is essential to maintain synaptic transmission during neuronal activity. Central to this is SV endocytosis, with three specific modes proposed to exist in central nerve terminals (Chanaday et al. 2019). The first is ultrafast endocytosis, which is dominant during sparse action potential stimulation (Watanabe et al. 2013, 2014). During high neuronal activity, activity‐dependent bulk endocytosis (ADBE) is the dominant mode (Clayton et al. 2008). Ultrafast endocytosis and ADBE both retrieve areas of plasma membrane to form endosomes, from which SVs bud to refill the recycling pool (Kokotos and Cousin 2015; Watanabe et al. 2014). Finally, clathrin‐mediated endocytosis (CME) is essential for the sorting and clustering of SV cargo for packaging into SVs (Granseth et al. 2006). Emerging molecular similarities between ultrafast endocytosis and ADBE suggest that they may be the same endocytosis mode (Imoto et al. 2022; Watanabe et al. 2013, 2018; Clayton et al. 2009; Kononenko et al. 2014; Soykan et al. 2017), with CME‐dependent cargo sorting displaced from the plasma membrane to presynaptic endosomes (Kononenko et al. 2014; Watanabe et al. 2014; López‐Hernández et al. 2022; Ivanova et al. 2021; Arpino et al. 2022).

Presynaptic dysfunction has been implicated as a contributing factor in HD (Milnerwood and Raymond 2010). In previous work we revealed a selective and activity‐dependent defect in SV cargo retrieval in striatal neurons derived from the *Htt* ^Q140/Q140^ knock‐in mouse (McAdam et al. 2020). This defect was only revealed during high‐intensity stimulation and specifically in striatal neurons suggesting that it may, at least in part, contribute to the degeneration of MSNs in HD in later life. Importantly, this defect resulted from a loss of htt function rather than a toxic gain of function of mutant htt (mhtt), suggesting an essential role for htt in SV recycling (McAdam et al. 2020). In this study, we determined how high intensity stimulation impacted the major endocytosis mode triggered during such activity—ADBE. We discovered that *Htt* ^Q140/Q140^ neurons have an intrinsic defect in triggering of ADBE that is conserved across all cell types examined. Furthermore, this defect is a result of a loss of htt function, since its depletion in *Htt* ^+/+^ neurons mimics the phenotype, with expression of exogenous htt in *Htt* ^Q140/Q140^ neurons able to restore physiological levels of ADBE. Increased ADBE was also observed in *Htt* ^Q140/+^ neurons, providing clinical relevance. Finally, removal of the mhtt allele in *Htt* ^Q140/+^ neurons did not correct the increase, confirming that haploinsufficiency was responsible for the phenotype.

## 2 Materials and Methods

### 2.1 Materials

Hydrophobically modified siRNA (hsiRNA) against htt (HTT10150) and a non‐targeting control (NTC) hsiRNA were based on a previously identified HTT functional targeting site (Alterman et al. 2015). The synthesis and purification of these hsiRNA were described in (McAdam et al. 2020). DNA encoding the fluorescent protein mCerulean (David Piston, Vanderbilt University (Rizzo et al. 2004)) was cloned into a N1 Clontech plasmid as described (Anggono et al. 2006). The plasmid pcDNA3.1 was from Invitrogen. Synaptophysin‐pHluorin (syp‐pH) was a gift from Prof. Leon Lagnado (University of Sussex). Htt expression plasmid (human Htt‐Q23‐pcDNA3.1, amino acids 1–3144, CHDI‐90002097), ZFP30645‐pAAV‐6P‐SWB‐Flag (CHDI‐90001335) and ZFP‐deltaDBD‐pAAV‐6P‐SWB‐FLAG (CHDI‐90001485) were provided by Cure Huntington's Disease Initiative Foundation via the Coriell Institute for Medical Research (Camden, NJ). Both AAV plasmids were packaged into AAV particles (serotype DJ) by Viagene Biotech (Tampa, USA).

The following antibodies were used: mouse anti‐Htt (1:5000; Merck MAB2166; RRID AB_2123255), rabbit anti‐SV2A (1:250; Abcam ab32942; RRID AB_778192) and mouse anti‐β‐actin (1:25000; Sigma‐Aldrich A3854; RRID AB_262011). Goat anti‐mouse secondary antibody (1:10000; 925–32 212; RRID AB_2716622) was purchased from LI‐COR Biosciences (Cambridge, UK) and donkey anti‐rabbit Alexa568 secondary antibody (1:1000, A10042; RRID AB_2534017) was purchased from Thermo Fisher Scientific. Tetramethylrhodamine (TMR)‐dextran (40 kDa), Neurobasal media, B‐27 supplement, penicillin/streptomycin, Minimal Essential Medium (MEM), Lipofectamine 2000, phosphate buffered salts were obtained from Life Technologies (Paisley, UK). AM1‐44 and Advasep‐7 were obtained from Cambridge Bioscience (Cambridge, UK). Glutaraldehyde and osmium tetroxide were from Agar Scientific (Essex, UK). Fluorsave was obtained from Merck (Gillingham, UK). All other reagents were obtained from Sigma‐Aldrich (Poole, UK).

### 2.2 Mouse Colony Maintenance and Management

*Htt* ^Q140/Q140^ knock‐in mice (which express a chimeric mouse/human exon 1 inserted into the murine htt gene containing a 140 CAG expansion (Menalled et al. 2003)) were maintained as homozygotes. Wild‐type C57BL/6J mice were sourced from an in‐house colony at the University of Edinburgh (original source—Charles River Laboratories, UK). *Htt* ^Q140/+^ offspring were generated by mating *Htt* ^+/+^ males and *Htt* ^Q140/Q140^ females. All mouse colonies were housed in standard open top caging on a 14 h light/dark cycle (light exposure between 07:00 and 21:00). Breeders were fed RM1 chow, whereas stock mice were maintained on RM3 chow. All mice had free access to water. Gene sequencing to confirm CAG repeat length in *Htt* ^Q140/Q140^ mice was performed by Laragen (Culver City, US).

All animal work was performed in accordance with the UK Animal (Scientific Procedures) Act 1986, under Project and Personal Licence authority and was approved by the Animal Welfare and Ethical Review Body at the University of Edinburgh (Home Office Project licence number 7008878 and PP5745138 to Prof. Cousin). All animals were killed by schedule 1 procedures in accordance with UK Home Office Guidelines. Specifically, pregnant adult (> 2 months old) females were killed either by cervical dislocation or exposure to rising CO_2_, followed by decapitation to confirm death. Embryos were killed by decapitation, followed by destruction of the brain. For embryonic culture preparations, one adult female mouse was time mated with a male stud. Once pregnancy was confirmed, the female was housed alone until culling when embryos were between E16 and E18. For postnatal culture preparations, the female was time mated and housed as above, with P5–7 pups culled by injection of pentobarbitone overdose, with death confirmed via the destruction of the brain. Total number of animals used for the study: dams = 70, embryos = 420, pups = 88.

### 2.3 Primary Neuronal Culture

Dissociated primary hippocampal and striatal enriched cultures from both sexes were generated from E16 to 18 mouse embryos (McAdam et al. 2020). Dissected tissue was pooled from multiple embryos of the same genotype and digested in papain (0.3 U/mL) supplemented PBS at 37°C for 20 min. Papain was then removed and replaced with DMEM/F12 (Dulbecco's Modified Eagle Medium: Nutrient Mixture F‐12) supplemented with 10% *w*/*v* foetal bovine serum and triturated to obtain a single‐cell suspension. The suspension was centrifuged for 5 min at 347 *g*. The supernatant was discarded and the pellet resuspended in Neurobasal medium supplemented with 2% B‐27 supplement, 0.5 mM L‐glutamine, and 1% *v*/*v* penicillin/streptomycin and then plated on 25 mm coverslips coated with poly‐d‐lysine in boric acid (100 mM, pH 8.5) with laminin spots. Hippocampal and striatal neurons were plated at a density of 4 × 10^4^ and 6.5 × 10^4^ cells/coverslip for imaging, respectively, and at 1.2 × 10^5^ cells/coverslip for lysis. After 1 h, coverslips were flooded in the same Neurobasal media as above, which was supplemented after 72 h with 1 μM cytosine β‐d‐arabinofuranoside to inhibit glial proliferation. In htt knockdown experiments, 0.5 μM of either hsiRNA or NTC was added to culture media after 7 days in vitro (DIV). All other transfections were performed after 7 or 8 DIV using 0.6–1.0 μg of DNA per plasmid and 2 μL of lipofectamine 2000 per well. For single cell TMR‐dextran imaging experiments, 1 μg of either pcDNA3.1 or Htt‐Q23‐pcDNA3.1 was co‐transfected with 0.8 μg of mCerulean‐N1 and 1.8 μL of lipofectamine 2000 per well. Imaging experiments were performed after 13–15 DIV.

Primary cultures of cerebellar granule neurons were prepared from the cerebella of 7‐day‐old mice of both sexes as previously described (Marland et al. 2016). The dissected cerebellum from multiple pups of the same genotype was pooled and digested with 0.025% trypsin in Ca^2+^/Mg^2+^ free Hanks Balanced Salt Solution supplemented with 10 mM HEPES pH 7.3 for 20 min at 37°C. Digested tissue was washed in Dulbecco's Modified Eagle Medium supplemented with 10% foetal bovine serum, 60 U/mL DNase and 1% penicillin/streptomycin, and triturated in the same medium to obtain a single cell suspension. Cells were diluted to the required density in Dulbecco's Modified Eagle Medium supplemented with 10% foetal bovine serum and 1% penicillin/streptomycin, and plated in 75 μL spots on 25 mm glass coverslips coated with 50 μg/mL poly‐d‐lysine. After 1 h, coverslips were flooded with Neurobasal medium supplemented with 2% B27, 0.5 mM L‐glutamine, an additional 20 mM KCl (total concentration 25 mM) and 1% penicillin/streptomycin. The following day the medium was further supplemented with 1 μM cytosine β‐D‐arabinofuranoside. Knockdown experiments were performed in an identical manner to hippocampal and striatal cultures with the exception that hsiRNA or NTC were added at 3 DIV, and imaged at 9 or 10 DIV. Cultures were transfected at 5 DIV and imaged at 9 or 10 DIV.

Primary hippocampal cultures from *Htt* ^+/+^ and *Htt* ^Q140/+^ mice were treated with adeno‐associated virus (AAV) containing zinc finger proteins which either selectively target the extended CAG repeat in the mutant *Htt* gene (C‐Term FLAG tagged, human mutant Htt‐repressor ZFP30645 (CHDI‐90001335)) or a control non‐targeting AAV with DNA binding ability deleted (CHDI‐90001485) directly into the culture medium at 7 DIV. AAVs were incubated with the neurons until they were required for experimentation at 14 DIV.

### 2.4 Western Blotting

Primary hippocampal cultures from *Htt* ^+/+^ and *Htt* ^Q140/+^ mice transduced with AAV‐containing zinc finger proteins were lysed in SDS sample buffer (67 mM SDS, 2 mM EGTA, 67 mM Tris, 9.3% glycerol, 12% β‐mercaptoethanol, bromophenol blue, pH 7.4). Lysates were boiled at 95°C for 10 min. Samples were loaded and resolved using 8% SDS‐PAGE gels using a Bio‐Rad Mini‐PROTEAN Tetra Vertical Electrophoresis Cell. Separated proteins were transferred to a nitrocellulose membrane using a Bio‐Rad Mini Trans‐Blot Cell transfer apparatus. The efficiency of protein transfer was assessed by staining transferred membranes with Ponceau Red. Ponceau staining was removed by briefly washing in TBST (20 mM Tris, 135 mM NaCl, 0.1% Tween 20, pH 7.6). Membranes were incubated for 1 h in blocking buffer (1:1 TBST:Intercept [TBS] Blocking Buffer) before overnight incubation with anti‐Htt antibody diluted in blocking buffer at 4°C. Membranes were washed 3 times for 30 min in TBST before and after 1 h incubation at room temperature in the secondary antibody diluted in blocking buffer. Protein bands were detected with an Odyssey M scanner (LI‐COR Biosciences) using Image Studio Lite software v5.2 and analyzed using FIJI. Experimental *n* represents individual coverslips.

### 2.5 Quantification of Nerve Terminal Number Using Syp‐pH

Nerve terminal numbers in individual neurons were visualized using syp‐pH. Coverslips containing primary cultures (striatal, hippocampal and cerebellar) were mounted in a Warner imaging chamber (RC‐21BRFS) embedded with parallel platinum wires (6 mm apart). Cultures were subjected to continuous perfusion in alkaline imaging buffer containing (in mM): 69 NaCl, 50 NH_4_Cl, 2.5 KCl, 2 CaCl_2_, 2 MgCl_2_, 25 HEPES, 30 glucose, 0.01 CNQX, and 0.05 AP5, pH 7.4 to reveal total pHluorin fluorescence. Imaging was performed on a Zeiss Axio Observer D1 inverted epifluorescence microscope (Zeiss Ltd. Germany) using a ×40 1.3 NA oil immersion objective at room temperature. Images were acquired at 4 s intervals using a Hamamatsu Orca‐ER camera (Hamamatsu, Japan). Wavelength settings were 480 nm excitation and > 525 nm emission. Offline processing was performed using Fiji is just ImageJ (FIJI) software (Schindelin et al. 2012) and the Time Series Analyser V3 plugin. Regions of interest (ROIs) of identical size were placed over axonal nerve terminals, and the length of the relevant neurite was measured using the Simple Neurite Tracer plugin. The number of nerve terminals per 100 μm of neurite was then calculated. In all cases, *n* refers to the number of independent coverslips examined.

### 2.6 TMR‐Dextran Uptake

TMR‐dextran uptake was performed as described previously (Marland et al. 2016). Neuronal cultures were placed into a Warner imaging chamber containing imaging buffer; and were stimulated with a train of 400 action potentials (40 Hz, 10 s) in the presence of TMR‐dextran (50 μM), which was washed away immediately after stimulation. Continuous perfusion with imaging buffer occurred throughout imaging using a 40× oil immersion objective at 550 nm excitation and > 575 nm emission. Images were thresholded using the Max‐Entropy algorithm and the number of puncta was counted using the Analyse Particles tool which selected puncta within the size range of 0.5–2.5 μm^2^ (both in FIJI). The average number of TMR‐dextran puncta per field of view for each experiment (7–10 fields per experiment) was averaged for the same conditions. The value for the unstimulated background in corresponding fields of an identical size was subtracted to give the corrected TMR‐dextran uptake value. At least four independent experiments were performed for each experimental condition. The TMR‐dextran uptake data in *Htt* ^+/+^ hippocampal neurons presented in Figure 5F, is also presented in Figure 7A since uptake experiments with *Htt* ^Q140/+^ hippocampal neurons were performed at the same time.

### 2.7 Electron Microscopy

Morphological analysis of bulk endosome generation was performed as described previously (Clayton et al. 2008). Cultured neurons were placed into imaging buffer and stimulated with 400 action potentials delivered at 40 Hz in the presence of 10 mg/mL horseradish peroxidase (HRP). HRP was then immediately washed off after stimulation and fixed using 2% glutaraldehyde in PBS at 37°C for 30 min. After washing in 100 mM Tris (pH 7.4), HRP was developed with 0.1% *w*/*v* 3,3′‐diaminobenzidine and 0.2% *v*/*v* hydrogen peroxide. After further washes in 100 mM Tris, cultures were stained with 1% *v*/*v* osmium tetroxide for 30 min. Samples were then dehydrated using an ethanol series and polypropylene oxide and embedded using Durcupan resin. Samples were sectioned, mounted on grids and viewed using an FEI Tecnai 12 transmission electron microscope (Oregon, USA). Acquired images were analysed in FIJI. HRP‐labelled structures with a diameter ≥ 100 nm were arbitrarily classified as endosomes. Typically, at least 10 fields of view were acquired for one coverslip of cells. The average number of endosomes per nerve terminal was calculated for each coverslip and represents the experimental *n*. Endosome size was determined by measuring the average diameter of endosomes using the measure tool in FIJI and calculated as the mean of two measurements: the longest axis of the endosome and its perpendicular width. Only endosomes with a clearly defined circumference were measured; those with large breaks in the membrane were excluded to avoid overestimating endosome size. The average size of the endosomes per field was calculated for each coverslip and represents the experimental *n*.

### 2.8 AM1‐44 Uptake

Hippocampal neurons were removed from culture medium and mounted into a Warner imaging chamber. Invaginating membrane was labeled with AM1‐44 (10 μM) diluted in imaging buffer by evoking SV turnover using electrical stimulation at 10 Hz for 30 s. Non‐internalized dye was washed from neurons three times using jskj1 mM ADVASEP‐7 diluted in imaging buffer. For all further steps, neurons were incubated in the dark. Neurons were fixed in 4% paraformaldehyde (PFA) diluted in PBS for 10 min before quenching PFA fluorescence with 50 mM NH_4_Cl diluted in PBS for 10 min. Neurons were then permeabilized with 0.01% Triton X‐100 diluted in PBS for 10 min before blocking with 5% Horse Serum and 0.2% BSA diluted in PBS for 1 h. Nerve terminals were identified by incubation with an SV2A primary antibody (1:250) diluted in block solution for 1 h at room temperature. Neurons were washed 3 times in PBS before incubation in donkey anti‐rabbit Alexa568 secondary antibody (1:1000) diluted in block solution for 1 h at room temperature. Cells were washed 3 times in PBS and once in double distilled water before mounting onto glass slides using FluorSave mounting medium, curing in the dark overnight at room temperature before storage at −20°C prior to imaging. Imaging was performed on an inverted Zeiss Axio Observer. Z1 microscope using EC Plan‐Neofluar 40× oil immersion objective (NA 1.3) and Colibri 7 LED light source (Zeiss). AM1‐44 was visualized at excitation 450–490 nm, beam splitter 495 nm and emission filter 500–550 nm; SV2A was visualized at excitation 538–562 nm, beam splitter 570 nm and emission filter 570–640 nm. The number of SV2A positive puncta per field of view was determined using FIJI plugin SynQuant (Wang et al. 2020)—an automated synapse detection algorithm–using a min/max particle size of 10/50 (roughly 0.5–2.5 μm^2^) and *z*‐score of 10. The number of SV2A positive puncta co‐localized with AM1‐44 was then determined by combining SV2A and AM1‐44 puncta using ‘intersect’ with the same inclusion criteria as above. The number of double positive ‘active’ synapses was then expressed as a percentage of the total number of SV2A puncta per field of view. Eight fields of view were analyzed per coverslip and a mean value generated. Experimental *n* represents individual coverslips derived from 3 independent primary neuronal preparations.

### 2.9 Experimental Design and Statistical Analysis

All statistical analysis was performed in Graph Pad Prism 6.0. The normality of the data distribution was assessed by the Shapiro–Wilk normality test with the significance level set at α = 0.05. All groups either passed this normality test or were too small for normality to be assessed. In the latter scenario, normality was assumed. For comparison between genotypes, a two‐tailed unpaired Student's *t* test was performed in all cases. A one‐way ANOVA with Tukey's post‐test was used to compare more than two groups, with a Bonferroni's multiple‐comparison post‐test performed to compare multiple groups. Asterisks refer to *p*‐values as follows: *p ≤ 0.05, **p ≤ 0.005, ***p ≤ 0.001. No randomisation was performed to allocate subjects in the study. In imaging experiments, the experimenter was not blinded to genotype during image acquisition but was blinded for analysis, with the exception of the analysis of HRP‐labelled endosomes. Effect size was not estimated. No test for outliers was conducted. Neither sample size calculation nor exclusion criteria were predetermined. The study was exploratory and was not pre‐registered. All data are presented as mean values ± standard error of the mean (SEM). Sample sizes and statistical tests are indicated in the figure legends and Tables 1 and 2.

**TABLE 1:** Collated table of statistical analysis.

	Genotype	Mean ± SEM	*n* = # of coverslips/*N* = # of neuronal preparations	Comparison	*p*	Statistical test
Figure 1						
Figure 1C	WT	114.9 ± 11.50	9/4	WT vs. Q140	0.0296	Unpaired *t* test
	Q140	185.6 ± 25.26	11/5			
Figure 1F	WT	6.257 ± 0.429	5/2	WT vs. Q140	0.6702	
	Q140	6.527 ± 0.434	5/2			
Figure 1G	WT	0.237 ± 0.003	3/2	WT vs. Q140	0.7815	
	Q140	0.234 ± 0.009	3/2			
Figure 1J	WT	223.8 ± 27.7	6/3	WT vs. Q140	0.0038	
	Q140	621.9 ± 97.7	7/3			
Figure 1M	WT	6.990 ± 0.185	5/2	WT vs. Q140	0.0510	
	Q140	6.266 ± 0.256	5/2			
Figure 1N	WT	0.234 ± 0.002	3/2	WT vs. Q140	0.0999	
	Q140	0.247 ± 0.006	3/2			
Figure 2						
Figure 2C	WT	136.8 ± 10.62	10/3	WT vs. Q140	0.0336	Unpaired *t* test
	Q140	238.9 ± 50.64	7/3			
Figure 2F	WT	5.801 ± 0.561	3/1	WT vs. Q140	0.415	
	Q140	6.633 ± 0.724	3/1			
Figure 2G	WT	0.286 ± 0.011	3/1	WT vs. Q140	0.3869	
	Q140	0.301 ± 0.011	3/1			
Figure 3						
Figure 3C	WT	100 ± 6.35	10/4	WT vs. Q140	0.8767	Unpaired *t* test
	Q140	101.20 ± 4.40	10/3			
Figure 3F	WT	100 ± 2.51	6/2	WT vs. Q140	0.5910	
	Q140	103.20 ± 4.94	7/2			
Figure 3I	WT	100 ± 6.65	13/4	WT vs. Q140	0.7261	
	Q140	82.14 ± 7.21	10/4			
Figure 4						
Figure 4G	WT	3778 ± 68	15/3	WT vs. Q140	0.1458	Unpaired *t* test
	Q140	3910 ± 57	15/3			
Figure 4H	WT	29.53 ± 1.18	15/3	WT vs. Q140	0.3553	
	Q140	30.97 ± 0.98	15/3			
Figure 5						
Figure 5E	WT NTC	100 ± 10.49	5/3	WT NTC vs. Q140 NTC	0.0211	One‐way ANOVA with Bonferroni's Multiple Comparison test
	Q140 NTC	151.0 ± 10.19	6/3			
	WT KD	144.7 ± 8.81	7/3	WT NTC vs. WT KD	0.0426	
	Q140 KD	147.4 ± 12.13	6/3	WT NTC vs. Q140 KD	0.0360	
Figure 5F	WT NTC	100 ± 10.37	16/3	WT NTC vs. Q140 NTC	< 0.0001	
	Q140 NTC	204.5 ± 17.32	14/3			
	WT KD	155.2 ± 9.31	14/3	WT NTC vs. WT KD	0.0372	
	Q140 KD	170.3 ± 18.32	13/3	WT NTC vs. Q140 KD	0.0048	
Figure 5G	WT NTC	100 ± 12.80	10/3	WT NTC vs. Q140 NTC	0.0455	
	Q140 NTC	150.1 ± 12.63	9/3			
	WT KD	171.2 ± 13.83	10/3	WT NTC vs. WT KD	0.0013	
	Q140 KD	171.9 ± 9.65	10/3	WT NTC vs. Q140 KD	0.0011	
Figure 6						
Figure 6E	WT Emp	100 ± 3.40	11/3	WT Emp vs. Q140 Emp	< 0.0001	One‐way ANOVA with Bonferroni's Multiple Comparison test
	Q140 Emp	184.6 ± 7.04	13/3	Q140 Emp vs. WT Q23‐htt	< 0.0001	
	WT Q23‐htt	98.9 ± 4.01	12/3	Q140 Emp vs. Q140 Q23‐htt	< 0.0001	
	Q140 Q23‐htt	113.2 ± 2.45	13/3			
Figure 7						
Figure 7A	WT	100 ± 10.37	16/3	WT vs. Het	0.0017	Unpaired *t* test
	Het	149.2 ± 7.83	21/3			
Figure 7C	Ø	1.0 ± 0.051	8/3	Ø vs. NTC	> 0.999	One‐way ANOVA with Bonferroni's Multiple Comparison test
	NTC	0.999 ± 0.092	5/3	Ø vs. KD	0.0002	
	KD	0.472 ± 0.077	5/3	NTC vs. KD	0.0006	
Figure 7F	NTC	100 ± 12.53	6/3	NTC vs. KD	0.846	Unpaired *t* test
	KD	104.2 ± 17.71	5/3			

**TABLE 2:** Collated table of advanced statistical analysis for ANOVA.

	Genotype/treatment	Mean ± SEM	*n* = # of coverslips/*N* = # of neuronal preparations	Degrees freedom (DF)	*F*‐value	*p*	Statistical test
Figure 5							
Figure 5E	WT NTC	100 ± 10.49	5/3	56	10.20	< 0.0001	One‐way ANOVA
	Q140 NTC	151.0 ± 10.19	6/3				
	WT KD	144.7 ± 8.81	7/3				
	Q140 KD	147.4 ± 12.13	6/3				
Figure 5F	WT NTC	100 ± 10.37	16/3	23	4.663	0.0125	
	Q140 NTC	204.5 ± 17.32	14/3				
	WT KD	155.2 ± 9.31	14/3				
	Q140 KD	170.3 ± 18.32	13/3				
Figure 5G	WT NTC	100 ± 12.80	10/3	38	7.698	0.0004	
	Q140 NTC	150.1 ± 12.63	9/3				
	WT KD	171.2 ± 13.83	10/3				
	Q140 KD	171.9 ± 9.65	10/3				
Figure 6							
Figure 6E	WT Emp	100 ± 3.40	11/3	48	78.40	< 0.0001	One‐way ANOVA
	Q140 Emp	184.6 ± 7.04	13/3				
	WT Q23‐htt	98.9 ± 4.01	12/3				
	Q140 Q23‐htt	113.2 ± 2.45	13/3				
Figure 7							
Figure 7C	Ø	1.0 ± 0.051	8/3	17	17.48	0.0001	One‐way ANOVA
	NTC	0.999 ± 0.092	5/3				
	KD	0.472 ± 0.077	5/3				

## 3 Results

### 3.1 Htt^Q140^ ^/Q140^ Neurons Display Excess ADBE

Htt is proposed to coordinate a series of molecular processes at a number of neuronal subcellular compartments, including the presynapse (Saudou and Humbert 2016). In agreement, we revealed a specific, activity‐dependent defect in SV cargo retrieval that was exclusive to striatal neurons derived from the *Htt* ^Q140/Q140^ knock‐in mouse (McAdam et al. 2020). Furthermore, this defect was due to loss of htt function, rather than a toxic gain of mhtt function, since (1) removal of htt from *Htt* ^+/+^ striatal neurons recapitulated the *Htt* ^Q140/Q140^ phenotype and (2) expression of htt with a non‐pathological polyglutamine repeat in *Htt* ^Q140/Q140^ striatal neurons fully restored normal function (McAdam et al. 2020).

**FIGURE 1:**
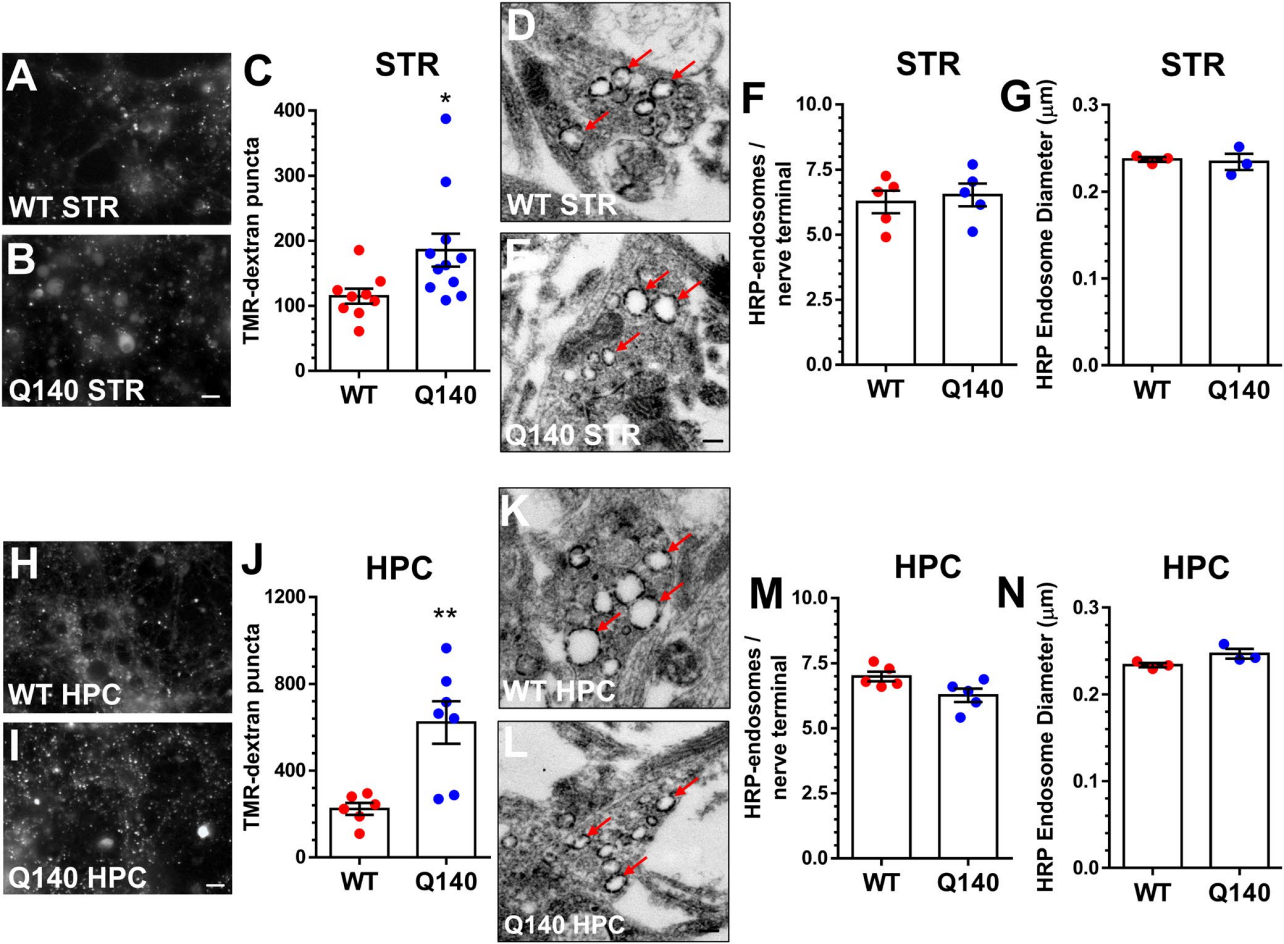
*Htt* ^Q140/Q140^ neurons from striatum and hippocampus display excess recruitment of ADBE. (A–C, H–J) Primary cultures of either striatal (STR, A–C) or hippocampal (HPC, H–J) neurons generated from either *Htt* ^+/+^ (WT) or *Htt* ^Q140/Q140^ (Q140) mice were challenged with a train of 400 action potentials (40 Hz) in the presence of 50 μM TMR‐dextran. TMR‐dextran was washed out immediately after stimulation. (A, B, H, I) Representative images of TMR‐dextran uptake from either WT STR (A), Q140 STR (B), WT HPC (H) and Q140 HPC (I) are displayed. Scale bar indicates 15 μm. (C, J) Quantification of evoked TMR‐dextran uptake ± SEM for either STR (**C**: WT *n* = 9, Q140 *n* = 11) or HPC (J: WT *n* = 6, Q140 *n* = 7). Two‐tailed Student's *t* test STR * = *p* = 0.0296, HPC ** = *p* = 0.0038. (D–G, K–N) Primary cultures of either striatal (STR, D–G) or hippocampal (HPC, K–N) neurons generated from either WT or Q140 mice were challenged with a train of 400 action potentials (40 Hz) in the presence of 10 mg/mL horse radish peroxidase (HRP). All cultures were fixed immediately after stimulation. (D, E, K, L) Representative images of HRP‐labelled nerve terminals from either WT STR (D), Q140 STR (E), WT HPC (K) and Q140 HPC (L) are displayed. Red arrows indicate examples of HRP‐labelled endosomes. Scale bar indicates 200 nm. (F, M) Quantification of the number of HRP‐labelled endosomes per nerve terminal ± SEM (*n* = 5 for all conditions). Two‐tailed Student's *t* test, STR *p* = 0.670, HPC *p* = 0.051. (G, N) Quantification of the average diameter of the HRP‐labelled endosomes for each genotype ± SEM (*n* = 3 for all conditions). Two‐tailed Student's *t* test, STR *p* = 0.7815, HPC *p* = 0.0999.

The activity‐dependence of the SV cargo retrieval phenotype described above was of particular relevance to HD, since repeated periods of high activity may render MSNs vulnerable to this physiological stressor. One SV endocytosis mode that is specifically triggered by periods of high activity is ADBE (Clayton et al. 2008). Therefore, we determined whether altered ADBE could be responsible for the activity‐dependent defect in SV cargo retrieval in *Htt* ^Q140/Q140^ striatal neurons. In these experiments, primary striatal neuronal cultures derived from either *Htt* ^Q140/Q140^ or *Htt* ^+/+^ embryos were challenged with a train of 400 action potentials (40 Hz). This stimulation protocol maximally triggers ADBE and is sufficient to reveal the SV cargo trafficking defect (Clayton et al. 2008; McAdam et al. 2020). During the stimulus, neurons were incubated with the large (40 kDa) fluid phase marker TMR‐dextran, which is selectively accumulated via this endocytosis mode. *Htt* ^+/+^ control neurons displayed a robust activity‐dependent uptake of this reporter (Figure 1A–C). Intriguingly, *Htt* ^Q140/Q140^ striatal cultures displayed a significant increase in the number of nerve terminals exhibiting TMR‐dextran uptake when compared to *Htt* ^+/+^ controls (Figure 1A–C). This suggested that ADBE was increased, rather than decreased, in this *Htt* ^Q140/Q140^ neuronal subtype.

TMR‐dextran uptake primarily reports how many nerve terminals perform ADBE, rather than the extent of ADBE at individual synapses (Cousin and Smillie 2021). Therefore, to determine whether ADBE is increased at individual synapses, we examined the generation of bulk endosomes within nerve terminals during high neuronal activity. This was achieved by monitoring the activity‐dependent uptake of a different fluid phase marker, HRP, followed by immediate fixation and conversion of HRP to an electron dense reaction product. This protocol allows the visualisation of the number of bulk endosomes formed from the plasma membrane during a stimulus train (Harper and Smillie 2021). When the number of HRP‐labelled bulk endosomes was compared between *Htt* ^+/+^ and *Htt* ^Q140/Q140^ striatal neurons, there was no significant difference between the two genotypes (Figure 1D–F). Additionally, there was no difference in the size of the endosomes generated by either genotype (Figure 1G). Taken together, these results suggest that ADBE is disproportionately recruited in striatal *Htt* ^Q140/Q140^ neurons during high activity, but the extent of ADBE at individual nerve terminals is unaffected.

The findings above suggest that *Htt* ^Q140/Q140^ striatal neurons may increase the number of nerve terminals performing ADBE during high activity to compensate for defective SV cargo retrieval. To determine this, we examined ADBE in neurons derived from the *Htt* ^Q140/Q140^ hippocampus, which do not display an activity‐dependent SV cargo retrieval defect (McAdam et al. 2020). As before, TMR‐dextran uptake was evoked by a train of 400 action potentials (40 Hz) in either *Htt* ^+/+^ or *Htt* ^Q140/Q140^ hippocampal cultures. Interestingly, hippocampal *Htt* ^Q140/Q140^ neurons also displayed a significant increase in the number of nerve terminals undergoing ADBE when compared to *Htt* ^+/+^ controls (Figure 1H–J). Furthermore, there was no difference in the number of bulk endosomes or the size of the bulk endosomes formed in hippocampal *Htt* ^Q140/Q140^ neurons when the HRP uptake assay was performed (Figure 1K–N). Therefore, the increase in ADBE occurring at individual nerve terminals does not appear to be an adaptation to dysfunctional SV cargo retrieval during high activity in *Htt* ^Q140/Q140^ striatal neurons.

**FIGURE 2:**
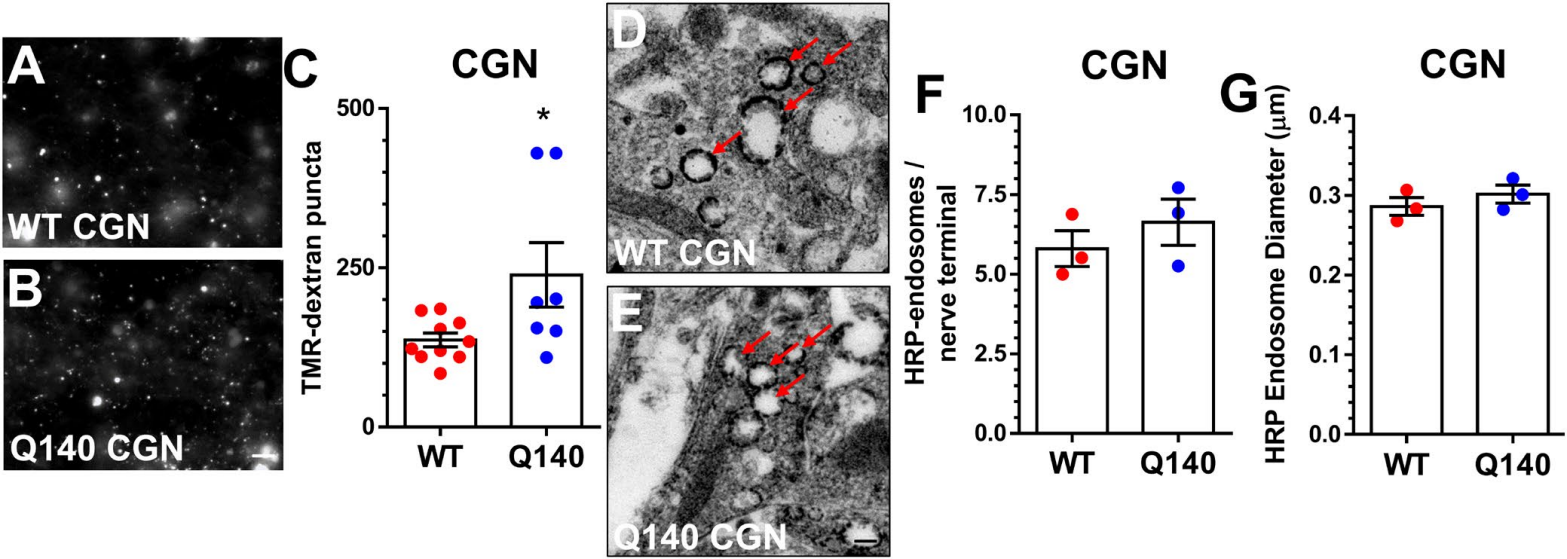
Cerebellar *Htt* ^Q140/Q140^ neurons display excess recruitment of ADBE. (A–C) Primary cultures of cerebellar granule neurons (CGN) from either *Htt* ^+/+^ (WT) or *Htt* ^Q140/Q140^ (Q140) mice were challenged with a train of 400 action potentials (40 Hz) in the presence of 50 μM TMR‐dextran. TMR‐dextran was washed out immediately after stimulation. (A, B) Representative images of TMR‐dextran uptake from WT CGN (A) and Q140 CGN (B) are displayed. Scale bar indicates 15 μm. (C) Quantification of evoked TMR‐dextran uptake ± SEM (C: WT *n* = 10, Q140 *n* = 7). Two‐tailed Student's *t* test, * = *p* = 0.0336. (D–G) Primary cultures of CGNs from either WT or Q140 mice were challenged with a train of 400 action potentials (40 Hz) in the presence of 10 mg/mL horse radish peroxidase (HRP). All cultures were fixed immediately after stimulation. (D, E) Representative images of HRP‐labelled nerve terminals from either WT CGN (D) or Q140 CGN (E) are displayed. Red arrows indicate examples of HRP‐labelled endosomes. Scale bar indicates 200 nm. (F) Quantification of the number of HRP‐labelled endosomes per nerve terminal ± SEM (*n* = 3 for both conditions). Two‐tailed Student's *t* test, *p* = 0.415. (G) Quantification of the average diameter of HRP‐labelled endosomes ± SEM (*n* = 3 for both conditions). Two‐tailed Student's *t* test, *p* = 0.3869.

The increased recruitment of ADBE during high activity in both striatal and hippocampal *Htt* ^Q140/Q140^ neurons suggests it may be a universal outcome of mhtt expression at central nerve terminals. To test this hypothesis, we analysed ADBE in a third neuronal subtype, cerebellar granule neurons. These excitatory neurons are the most numerous in the mammalian brain and routinely receive high frequency input from mossy fibre terminals (Burgoyne and Cambray‐Deakin 1988). To determine whether *Htt* ^Q140/Q140^ cerebellar neurons displayed a similar ADBE defect to both hippocampal and striatal neurons, we examined activity‐dependent uptake of TMR‐dextran in primary cerebellar cultures from either *Htt* ^+/+^ and *Htt* ^Q140/Q140^ mice. When this experiment was performed, a significant increase in the number of TMR‐dextran positive nerve terminals in *Htt* ^Q140/Q140^ cerebellar neurons was observed compared to *Htt* ^+/+^ (Figure 2A–C). Similar to striatal and hippocampal *Htt* ^Q140/Q140^ neurons, there was no significant change in the number or size of HRP‐labelled bulk endosomes formed during high activity in *Htt* ^Q140/Q140^ cerebellar neurons (Figure 2D–G). Therefore, across three independent cellular subtypes, *Htt* ^Q140/Q140^ neurons display an increased recruitment of ADBE at nerve terminals.

**FIGURE 3:**
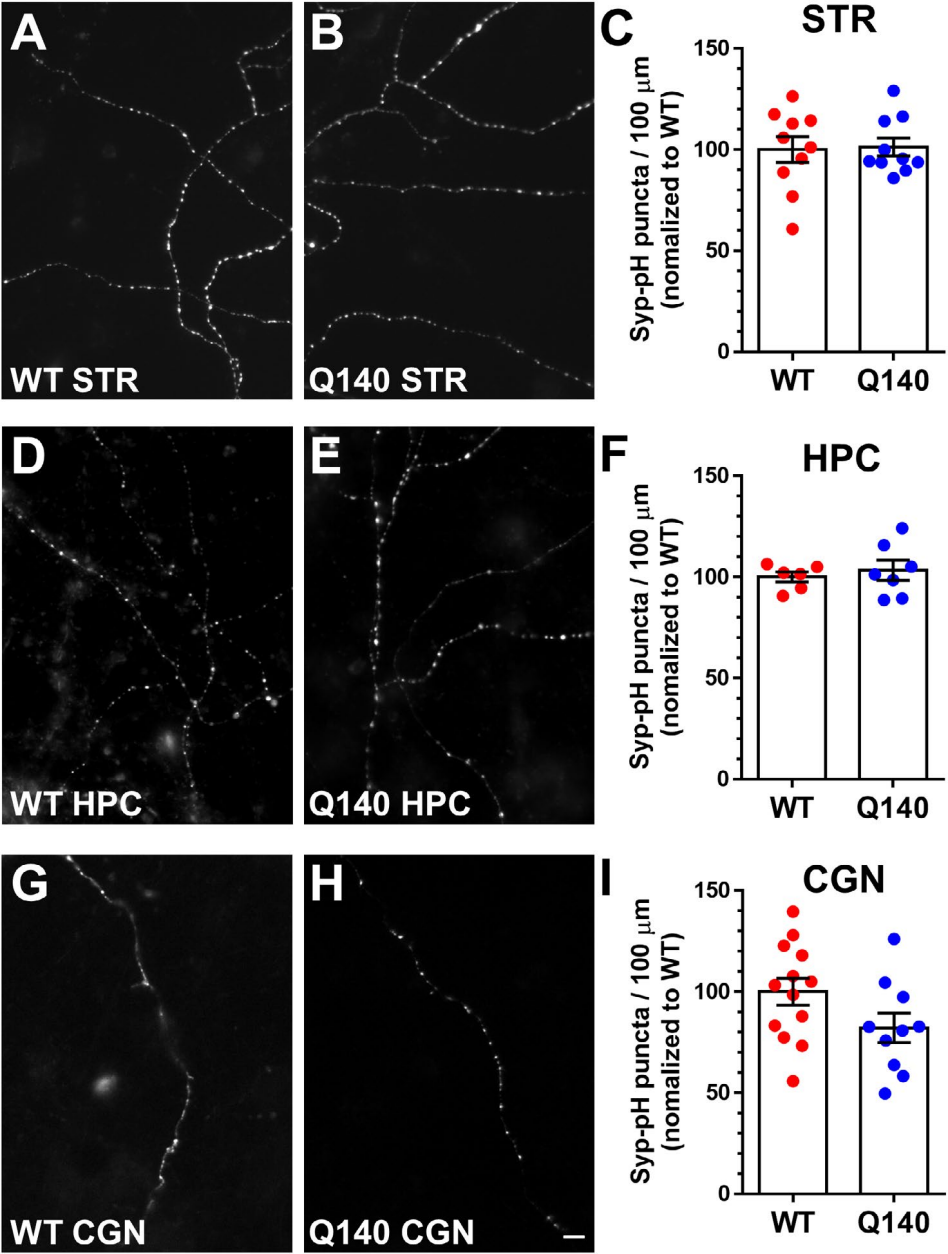
*Htt* ^Q140/Q140^ neurons do not have more nerve terminals. Primary cultures of either striatal (STR, A–C) hippocampal (HPC, D–F) or cerebellar (CGN, G–I) neurons from either *Htt* ^+/+^ (WT) or *Htt* ^Q140/Q140^ (Q140) mice were transfected with synaptophysin‐pHluorin (syp‐pH). Neurons were challenged with an alkaline buffer to dequench the syp‐pH signal and reveal all nerve terminals. Representative images of both WT and Q140 STR (A, B), HPC (D, E) and CGN (G, H) neurites are displayed, scale bar = 5 μm. Quantification of the number of syp‐pH puncta per 100 μm of neurite is presented ± SEM for STR (C, WT *n* = 10, Q140 *n* = 10), HPC (F, WT *n* = 6, Q140 *n* = 7) and CGN (I, WT *n* = 13, Q140 *n* = 10). Two‐tailed Student's *t* test, STR *p* = 0.877, HPC *p* = STR *p* = 0.591, CGN *p* = 0.726.

**FIGURE 4:**
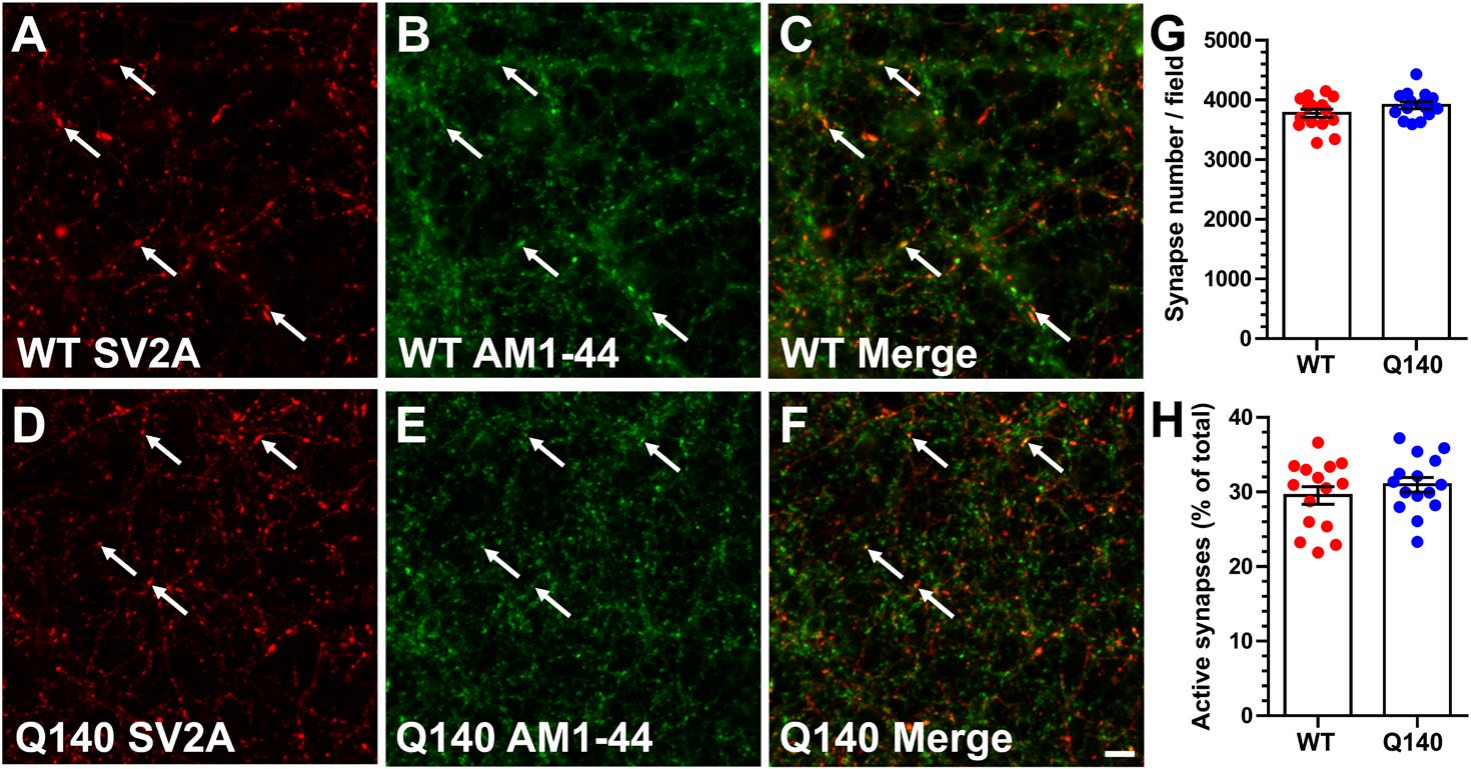
*Htt* ^Q140/Q140^ neurons do not have more active nerve terminals. Primary hippocampal cultures from either *Htt* ^*+/+*^ (WT) or *Htt* ^Q140/Q140^ (Q140) embryos were challenged with a train of 300 action potentials (10 Hz) in the presence of 10 μM AM1‐44. Non‐internalised AM1‐44 was immediately washed away following stimulation. Neurons were fixed and co‐stained for the SV marker SV2A. (A–F) Representative images of SV2A staining (A, D), AM1‐44 uptake (B, E) and merged images (C, F) from WT (A–C) and Q140 (D–F) neurons. White arrows represent AM1‐44 uptake in SV2A‐labelled synapses. Scale bar indicates 25 μm. (G) Quantification of the number of SV2A labelled synapses per field ± SEM (both conditions, *n* = 15). Two‐tailed Students' *t* test, *p* = 0.1458. (H) Quantification of number of active synapses (AM1‐44‐positive SV2A puncta) presented as a percentage of the total number of SV2A positive synapses ± SEM (both conditions, *n* = 15). Two‐tailed Students' *t* test, *p* = 0.3553.

An alternative explanation for the increased recruitment of ADBE observed in *Htt* ^Q140/Q140^ neurons from three independent brain regions was that there was a parallel increase in the number of nerve terminals. To address this point, we exploited the genetically encoded fluorescent reporter syp‐pH (Granseth et al. 2006). Syp‐pH has a pH‐sensitive GFP moiety (pHluorin) fused to an intraluminal loop of the SV protein synaptophysin, localising it to SVs. The pHluorin signal is quenched by the acidic interior of the SV; however, it is fully unquenched at pH 7 (Egashira et al. 2015). Therefore, the number of nerve terminals present in either *Htt* ^+/+^ or *Htt* ^Q140/Q140^ neurons was revealed via incubation with imaging buffer supplemented with NH_4_Cl. This manoeuvre neutralises all acidic compartments, thus dequenching syp‐pH fluorescence at individual nerve terminals, allowing quantification of the number of nerve terminals per 100 μm of neurite. When this analysis was performed, there was no significant difference in the number of syp‐pH puncta per 100 μm of neurite between genotypes for cultures derived from any brain region (Figure 3). This approach, however, quantifies synapses on individual neurites, and thus we additionally investigated whether there was any alteration to the overall synapse density. Hippocampal neurons from *Htt* ^+/+^ or *Htt* ^Q140/Q140^ mice were immunolabelled with an antibody against the SV protein SV2A, which is ubiquitously expressed in all synapses (Bajjalieh et al. 1994). Quantification of SV2A staining revealed no difference in overall synapse density (Figure 4A,D,G) between the genotypes, confirming that the increase in TMR‐dextran uptake in *Htt* ^Q140/Q140^ neurons is due to an increased recruitment of ADBE.

The increased number of synapses undergoing ADBE in *Htt* ^Q140/Q140^ neurons may reflect an increase in their intrinsic baseline activity culminating in more TMR‐dextran uptake. To address this, we monitored SV turnover using the styryl dye AM1‐44, which is an amphipathic dye which becomes fluorescent when inserted into the outer leaflet of the plasma membrane, thus reporting activity‐dependent SV endocytosis. To determine whether *Htt* ^Q140/Q140^ neurons display increased intrinsic excitability, AM1‐44 uptake was stimulated under conditions that do not trigger ADBE (300 action potentials delivered at 10 Hz, (Clayton et al. 2008)). Hippocampal neurons were immediately fixed following this stimulation protocol and were stained with antibodies against SV2A to identify nerve terminals (Figure 4A–F). Quantification of the number of SV2A puncta labelled with AM1‐44 revealed no different between *Htt* ^+/+^ and *Htt* ^Q140/Q140^ cultures (Figure 4H), confirming that the increased recruitment of ADBE in *Htt* ^Q140/Q140^ was not due to a difference in their intrinsic baseline activity. Therefore, taken together, the increase in activity‐dependent TMR‐dextran puncta in *Htt* ^Q140/Q140^ neurons is due to excess recruitment of ADBE.

### 3.2 Increased Recruitment of ADBE Results From Loss of *Htt* Function

We next determined whether the increased recruitment of ADBE across all *Htt* ^Q140/Q140^ cell types was due to loss of wild‐type htt function. This was achieved using hsiRNA that depletes both wild‐type htt and mhtt to the same extent in either *Htt* ^+/+^ or *Htt* ^Q140/Q140^ neurons respectively (McAdam et al. 2020; Alterman et al. 2015). The rationale for these experiments is that if increased recruitment of ADBE occurred via a toxic gain of mhtt function, depletion of mhtt in *Htt* ^Q140/Q140^ neurons should restore TMR‐dextran uptake to *Htt* ^+/+^ levels. Conversely, if loss of wild‐type htt function was responsible, depletion of htt in *Htt* ^+/+^ neurons would increase ADBE to that observed in *Htt* ^Q140/Q140^ neurons.

**FIGURE 5:**
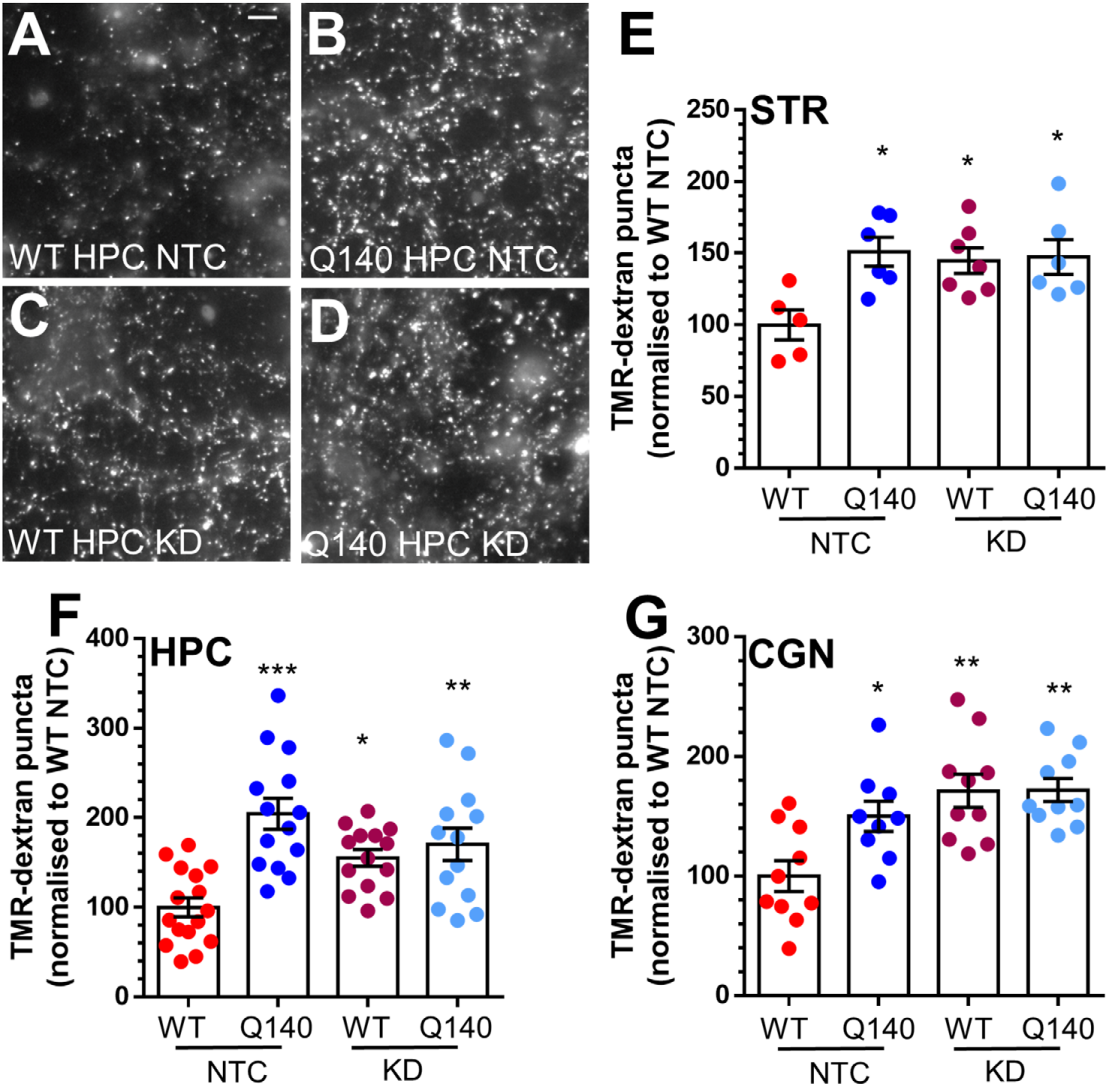
Depletion of htt increases ADBE in *Htt* ^+/+^ neurons but not in *Htt* ^Q140/Q140^ neurons. Primary cultures of either striatal (STR), hippocampal (HPC) or cerebellar (CGN) neurons generated from either *Htt* ^+/+^ (WT) or *Htt* ^Q140/Q140^ (Q140) mice were treated with 0.5 μM of htt hsiRNA (KD) or non‐targeting control (NTC) for 7 days. They were then challenged with a train of 400 action potentials (40 Hz) in the presence of 50 μM TMR‐dextran. TMR‐dextran was washed away immediately after stimulation. (A–D) Representative images of TMR‐dextran uptake from HPC neurons is displayed (A, WT NTC), (B, Q140 NTC), (C, WT KD) and (D, Q140 KD). Scale bar = 15 μm. (E–G) Quantification of evoked TMR‐dextran uptake ± SEM in either STR (E), HPC (F) and CGN (G) normalised to the WT NTC condition. (E, STR WT NTC *n* = 5, Q140 NTC *n* = 6, WT KD *n* = 7, and Q140 KD *n* = 6; F HPC WT NTC *n* = 16, Q140 NTC *n* = 14, WT KD *n* = 14, Q140 KD *n* = 13 G, CGN WT NTC *n* = 10, Q140 NTC *n* = 10, WT KD *n* = 9, Q140 KD *n* = 10). One‐way ANOVA, *** = *p* < 0.001, ** = *p* < 0.01; * = *p* < 0.05 when compared to WT NTC.

The impact of htt and mhtt depletion on ADBE was determined in *Htt* ^+/+^ and *Htt* ^Q140/Q140^ neurons derived from the hippocampus, striatum and cerebellum via treatment with either hsiRNA or a non‐targeting control (NTC). *Htt* ^Q140/Q140^ neurons from all three subtypes displayed a significant increase in the number of nerve terminals accumulating TMR‐dextran in comparison to *Htt* ^+/+^ when treated with NTC hsiRNA, in agreement with previous results (Figure 5). Importantly, when mhtt was depleted in *Htt* ^Q140/Q140^ neurons using hsiRNA, the number of nerve terminals that displayed activity‐dependent TMR‐dextran uptake was unchanged in relation to *Htt* ^Q140/Q140^ neurons incubated with NTC hsiRNA (Figure 5). This suggests that the loss of htt function was responsible for the increased recruitment of ADBE observed in *Htt* ^Q140/Q140^ neurons. To confirm this, htt was depleted in *Htt* ^+/+^ cultures using hsiRNA and compared to *Htt* ^+/+^ cultures treated with NTC hsiRNA. In htt‐depleted *Htt* ^+/+^ neurons from all three brain regions, there was a marked and significant increase in the number of nerve terminals displaying activity‐dependent TMR‐dextran uptake when compared to the NTC *Htt* ^+/+^ neurons (Figure 5). Therefore, the increased recruitment of ADBE during high activity in *Htt* ^Q140/Q140^ neurons is due to the loss of wild‐type htt function.

### 3.3 *Htt* Expression Rescues ADBE Defect in *Htt* ^Q140^ ^/Q140^ Neurons

**FIGURE 6:**
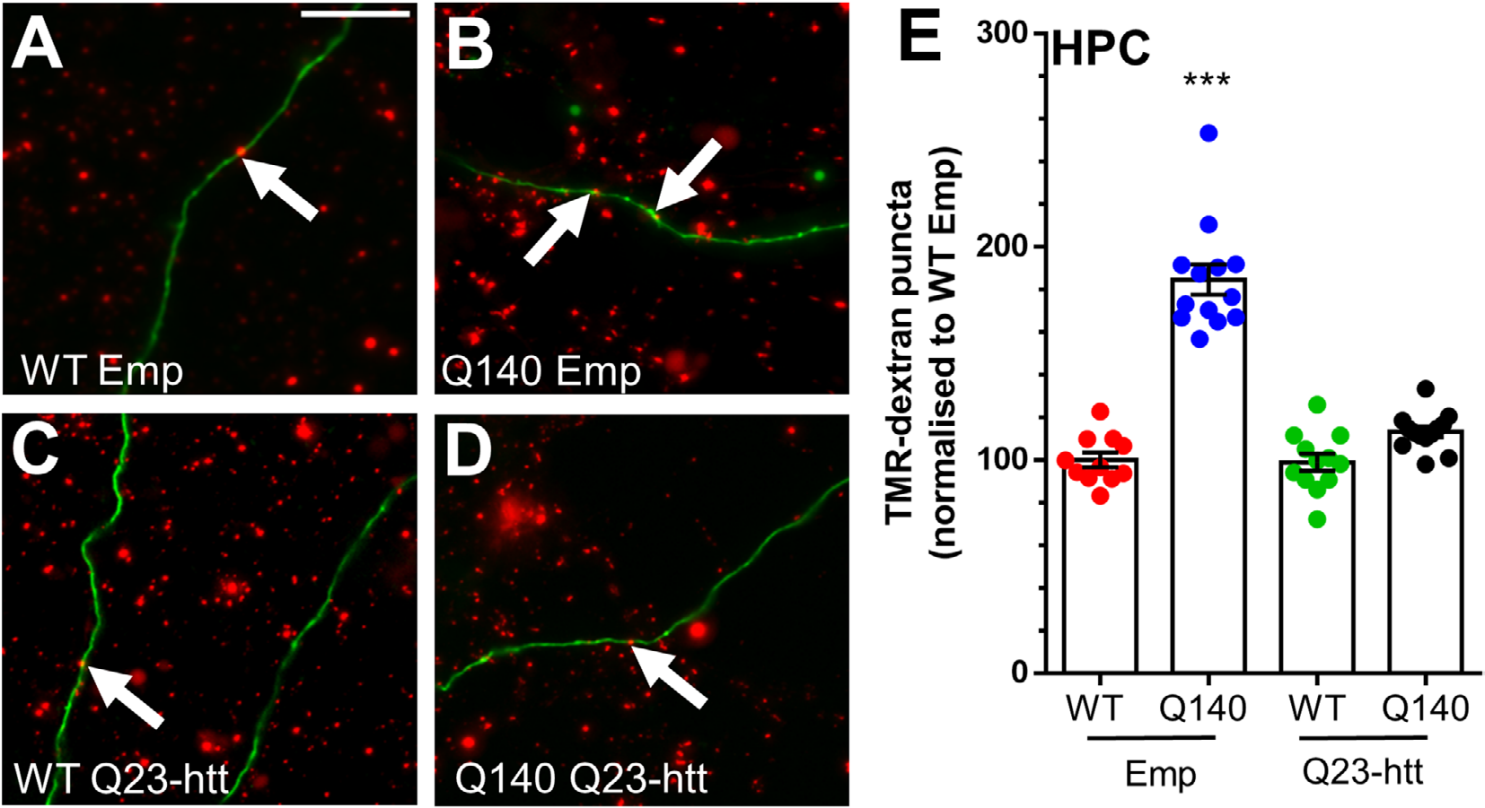
Correction of excess ADBE in *Htt* ^Q140/Q140^ neurons via expression of wild‐type htt. Primary cultures of hippocampal neurons generated from either *Htt* ^+/+^ (WT) or *Htt* ^Q140/Q140^ (Q140) mice were co‐transfected with either Q23‐htt or an empty pcDNA3.1 vector (Emp) and mCerulean to mark transfected neurons. After 7 days they were challenged with a train of 400 action potentials (40 Hz) in the presence of 50 μM TMR‐dextran. TMR‐dextran was washed away immediately after stimulation. (A‐D) Representative images of TMR‐dextran uptake are displayed (A, WT Emp), (B, Q140 Emp), (C, WT Q23‐htt) and (D, Q140 Q23‐htt). White arrows indicate TMR‐dextran uptake on individual neurites. Scale bar = 15 μm. (E) Quantification of evoked TMR‐dextran uptake ± SEM normalised to the WT Emp condition. (WT Emp *n* = 11, Q140 Emp *n* = 12, WT Q23‐htt *n* = 13, Q140 Q23‐htt *n* = 13). One‐way ANOVA, *** = *p* < 0.0001, when compared to WT Emp.

To confirm that loss of wild‐type htt function in *Htt* ^Q140/Q140^ neurons results in increased recruitment of ADBE, we determined whether this phenotype could be corrected via the introduction of htt with a non‐pathological polyglutamine repeat. To achieve this, we expressed htt with a polyglutamine tract of 23, Q23‐htt in both *Htt* ^+/+^ and *Htt* ^Q140/Q140^ hippocampal neurons and examined TMR‐dextran uptake during high activity (Figure 6A–D). When *Htt* ^+/+^ and *Htt* ^Q140/Q140^ neurons expressing an empty vector control (pcDNA3.1) were compared, a significant increase in the number of nerve terminals performing ADBE was again observed in *Htt* ^Q140/Q140^ neurons (Figure 6E). In *Htt* ^+/+^ neurons expressing exogenous Q23‐htt, there was no significant change in the number of TMR‐dextran puncta when compared to *Htt* ^+/+^ neurons expressing empty vector control (Figure 6E). Therefore, overexpression of exogenous Q23‐htt had no deleterious impact on ADBE. In contrast, expression of Q23‐htt in *Htt* ^Q140/Q140^ neurons resulted in a restoration in the number of nerve terminals displaying activity‐dependent TMR‐dextran uptake comparable to that observed in *Htt* ^+/+^ neurons (Figure 6E). Therefore, the increased recruitment of ADBE in *Htt* ^Q140/Q140^ neurons can be fully corrected by the introduction of Q23‐htt, confirming that this phenotype is due to a loss of wild‐type htt function.

### 3.4 *Htt* Haploinsufficiency Is Responsible for Increased Recruitment of ADBE

**FIGURE 7:**
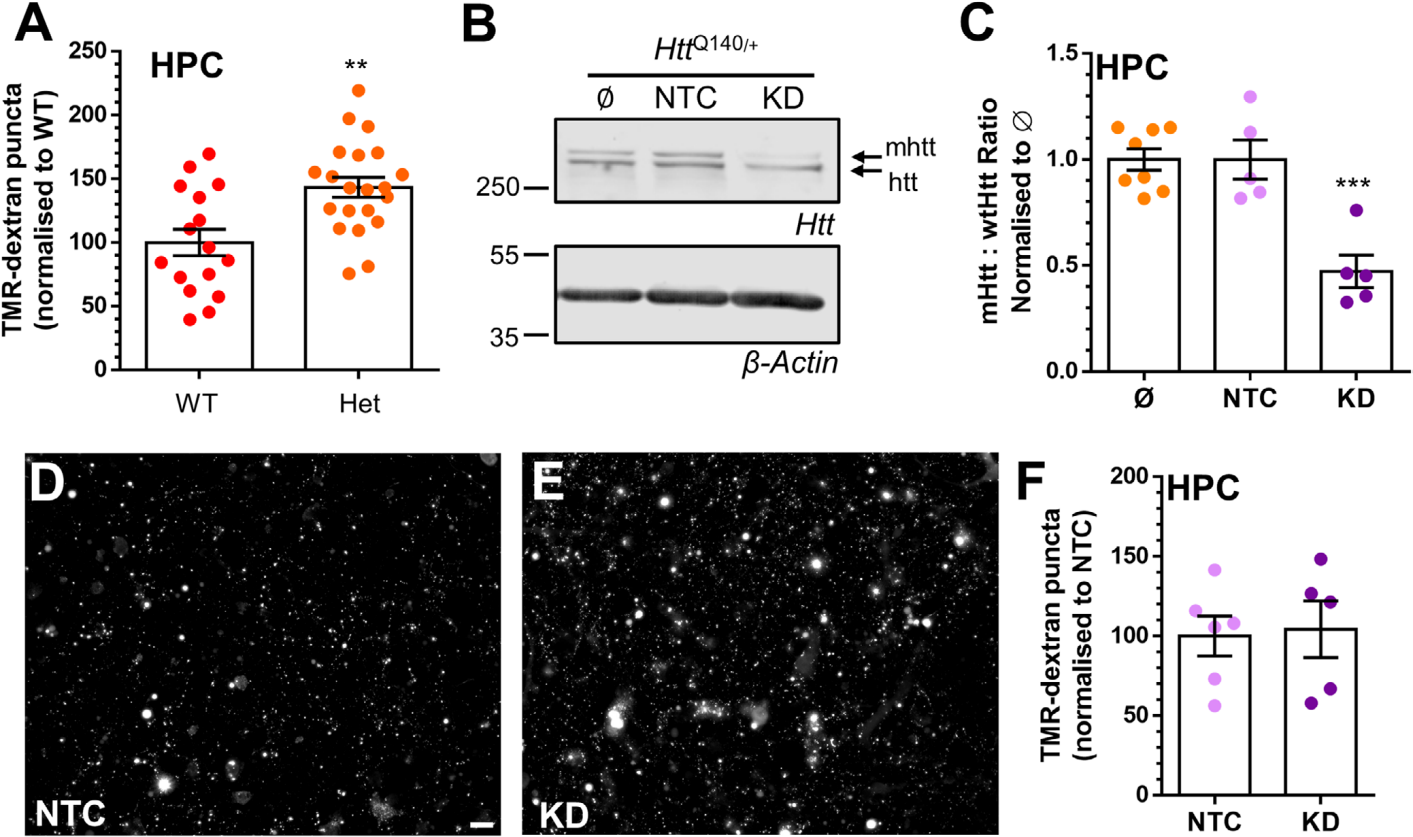
*Htt* ^Q140/Q140^ neurons display excess ADBE due to htt haploinsufficiency. (A) Primary cultures of hippocampal (HPC) neurons generated from *Htt* ^+/+^ (WT) or *Htt* ^Q140/+^ (Het) mice were challenged with a train of 400 action potentials (40 Hz) in the presence of 50 μM TMR‐dextran. TMR‐dextran was washed away immediately after stimulation. Quantification of evoked TMR‐dextran uptake ± SEM normalised to WT is displayed. (WT *n* = 16, Het *n* = 21). Unpaired *t* test *p* = 0.0017. (B–F) Primary cultures of *Htt* ^Q140/+^ HPC neurons were transduced with ZFP‐AAVs that either targeted the mhtt allele (KD) or not (NTC) at 7 DIV. (B, C) At 14 DIV neurons were lysed and analysed for the presence of htt and mhtt by Western blotting. Representative Western blots display htt and mhtt levels in untreated (Ø), NTC, and KD in addition to β‐actin. (C) Quantification of the ratio of mhtt to htt after normalisation to both actin and Ø (Ø *n* = 8, NTC *n* = 5, KD *n* = 5) One‐way ANOVA, *** = *p* < 0.0001, when compared to Ø. (D–F) At 14 DIV neurons were challenged with a train of 400 action potentials (40 Hz) in the presence of 50 μM TMR‐dextran. TMR‐dextran was washed away immediately after stimulation. (D, E) Representative images of TMR‐dextran uptake are displayed (D, NTC) and (E, KD). Scale bar = 15 μm. (F) Quantification of evoked TMR‐dextran uptake ± SEM normalised to the NTC condition. (NTC *n* = 6, KD *n* = 5). Unpaired Student's *t* test *p* = 0.846.

Individuals with HD typically have only one copy of the mhtt allele (Tyebji and Hannan 2017). Therefore, we determined whether the ADBE phenotype also occurred in *Htt* ^Q140/+^ neurons. To address this, we monitored activity‐dependent TMR‐dextran uptake in hippocampal *Htt* ^Q140/+^ neurons in comparison to *Htt* ^+/+^ neurons. When this experiment was performed, *Htt* ^Q140/+^ neurons displayed a significant increase in activity‐dependent TMR‐dextran uptake in relation to *Htt* ^+/+^ neurons (Figure 7A). Therefore, the increased activity‐dependent recruitment of ADBE also occurs in the heterozygous condition, suggesting that it may be clinically relevant.

The increased recruitment of ADBE in *Htt* ^Q140/+^ neurons (Figure 7A), and in *Htt* ^+/+^ neurons where wild‐type htt was depleted (Figure 5) suggest that this phenotype may be a result of htt haploinsufficiency. To determine this directly, we examined the impact on the activity‐dependent recruitment of ADBE in *Htt* ^Q140/+^ neurons where only mhtt was removed. To achieve this, we exploited an AAV which expresses zinc finger proteins (ZFP) that selectively target the extended CAG repeat of mhtt (Zeitler et al. 2019). We first determined the efficacy of mhtt depletion in *Htt* ^Q140/+^ hippocampal neurons transduced with AAVs containing the ZFP targeting mhtt or NTC ZFPs via western blotting. Two bands of approximately 350 kDa were detectable in lysates from *Htt* ^Q140/+^ neurons treated with the NTC, representing either htt or mhtt (Figure 7B,C). However, in lysates from *Htt* ^Q140/+^ neurons transduced with mhtt‐targeting ZFPs, mhtt was almost completely absent, with no discernable effect on wild‐type htt expression (Figure 7B,C).

After confirming the specificity of mhtt depletion, we determined whether transducing hippocampal *Htt* ^Q140/+^ neurons with either mhtt‐targeting or control ZFPs impacted the observed increase in ADBE. Transduced *Htt* ^Q140/+^ neurons were challenged with a train of 400 action potentials delivered at 40 Hz in the presence of TMR‐dextran as before. When the number of nerve terminals that accumulated this reporter was determined, there was no difference between *Htt* ^Q140/+^ neurons that retained both wild‐type htt and mhtt alleles, and those where the mhtt was depleted by the ZFP intervention (Figure 7D–F). Therefore, in neurons that replicate the genetic landscape of HD, the activity‐dependent increase in ADBE recruitment is due to htt haploinsufficiency, rather than toxic gain of function of the mhtt allele.

## 4 Discussion

We have identified an intrinsic defect in a specific presynaptic endocytosis mode (ADBE) in neurons derived from a pre‐symptomatic HD mouse model (the *Htt* ^Q140/Q140^ mouse). The defect manifests as an over‐recruitment of ADBE to synapses, rather than an increase in its extent at any given synapse. Importantly, this phenotype was due to a loss of htt function rather than a toxic gain of mhtt function, since (1) depletion of htt in *Htt* ^Q140/Q140^ neurons recapitulated the ADBE phenotype and (2) expression of htt with a non‐pathological polyglutamine repeat fully restored typical ADBE in *Htt* ^Q140/Q140^ neurons. Importantly, the ADBE defect occurred in *Htt* ^Q140/+^ neurons, demonstrating clinical relevance. Finally, removal of the mutant Q140 allele from these heterozygous neurons has no impact on excess ADBE recruitment, providing conclusive evidence that loss of htt function is mediating this phenotype via haploinsufficiency. This newly identified disease signature provides additional evidence that presynaptic dysfunction early in life may contribute to defects in both early development and later neurodegeneration in HD.

All neuronal subtypes derived from *Htt* ^Q140/Q140^ mice display an over‐recruitment of ADBE. Therefore, this phenotype is distinct from the activity‐ and striatal‐specific defect in SV cargo retrieval previously observed in *Htt* ^Q140/Q140^ and *Htt* ^Q140/+^ neurons (McAdam et al. 2020). Indeed, genetically‐encoded reporters of SV cargo retrieval (pHluorins) report no genotype difference in either hippocampal (McAdam et al. 2020) or cerebellar (K.J.S., unpublished data) *Htt* ^Q140/Q140^ neurons. This divergence between pHluorins and TMR‐dextran is not a result of the exclusion of SV cargo proteins from ADBE, since fully functional SVs form directly from bulk endosomes to repopulate the recycling SV pool (Cheung et al. 2010; Cheung and Cousin 2012, 2013, 2019). Futhermore, purified bulk endosomes contain the full inventory of SV cargo molecules (Kokotos et al. 2018). Additionally, antibody tagging of endogenous SV cargo proteins revealed their accumulation via ADBE (Okamoto et al. 2016). Intriguingly, pHluorin reporters are not accumulated in similar experiments (Nicholson‐Fish et al. 2015). Furthermore, interventions that disrupt ADBE do not impact exogenously expressed pHluorin reporters (Nicholson‐Fish et al. 2015; Bonnycastle et al. 2022, 2025; Blumrich et al. 2023). The most likely explanation for this discrepancy is that the TMR‐dextran assay provides a binary, rather than quantitative, output of the number of nerve terminals performing ADBE, whereas the pHluorin assay provides the extent of SV cargo retrieval at individual nerve terminals.

We recently revealed a developmental delay in ADBE recruitment in neurons that model Fragile X syndrome (Kim et al. 2024). However, this over‐recruitment is unlikely to reflect an accelerated maturation of *Htt* ^Q140/Q140^ or *Htt* ^Q140/+^ neurons, since no alterations in intrinsic excitability was observed when AM1‐44 uptake was examined. In further support, we observed no difference in the rate of neurite outgrowth, complexity of the developing arbor or the density of synapses through development in primary neuronal cultures from *Htt* ^Q140/+^ and *Htt* ^+/+^ mice (K.J.S., unpublished observations), suggesting the increased recruitment in ADBE is not a consequence of altered development.

Importantly, the excess recruitment of ADBE occurred in cultures enriched in inhibitory MSNs (striatal), low release probability excitatory neurons (hippocampal) and excitatory neurons adapted for high frequency input (cerebellar). Therefore, we propose that this phenotype is a universal consequence of htt loss of function. In support of this conclusion, *Htt* ^Q140/+^ neurons display over‐recruitment of ADBE, which cannot be corrected by the selective removal of the mhtt allele in these neurons. Therefore, haploinsufficiency of htt appears to be responsible for the ADBE phenotype in *Htt* ^Q140/+^ neurons, suggesting relevance for disease progression. Furthermore, HD‐like degeneration is a consequence of conditional knockout of htt in the adult mouse brain (Dragatsis et al. 2000). Additionally, *Htt* ^+/−^ mice present with a series of cognitive, motor, and pathological alterations that are very similar to knock‐in models of HD (Nasir et al. 1995; O'Kusky et al. 1999; Menalled et al. 2009). In contrast, our studies were conducted in the *Htt* ^Q140^ mouse, which is a well‐established model for studying HD, with a slow development of symptoms and replication of HD pathology observed in other HD mouse models (Menalled et al. 2003). It would be of definite future interest to investigate any convergence of SV recycling defects in other HD mouse models with a different genetic basis (e.g., the R6/2 N‐terminal over‐expression mouse) (Chang et al. 2015). However, a more interesting study would be to investigate SV recycling in mice where the *Htt* gene can be conditionally ablated (Dragatsis et al. 2000) to interrogate our haploinsufficiency hypothesis.

Htt interacts with many proteins in the presynapse (Harjes and Wanker 2003) and indeed aggregate‐induced disruption of endocytic function has been reported in several models (Meriin et al. 2003; Yu et al. 2014; Singh et al. 2024). However, our data suggest that htt acts as a repressor of ADBE triggering, but does not form part of the essential ADBE machinery. This is principally because loss of htt function does not influence the extent of ADBE, only its recruitment. What role(s) could htt perform to fulfill this function? One potential mechanism may relate to brain‐derived neurotrophic factor (BDNF). This is because its expression is reduced at both the mRNA and protein level in the brains of HD patients and in a series of HD mouse models (Zuccato and Cattaneo 2014). Importantly, BDNF is a key negative regulator of ADBE (Smillie et al. 2013), with protein kinase signalling cascades downstream from BDNF such as Akt and GSK3 also required to repress this pathway (Smillie and Cousin 2012; Clayton et al. 2010). In support, Akt1 is de‐enriched on bulk endosomes derived from *Htt* ^Q140/Q140^ cerebellar neurons (MAC and KJS unpublished observations). Therefore, it will be critical to determine whether BDNF delivery or modulation of its downstream signalling can correct the observed excess recruitment of ADBE in *Htt* ^Q140/+^ neurons. In a related point, the small GTPase Rac1 operates downstream from a number of growth factors to control actin‐dependent membrane remodelling (Costa et al. 2020). Actin assembly is also essential for ADBE (Wu et al. 2016; Soykan et al. 2017), specifically actin nucleation via both the formins mDia1/3 and the Rac1 signalling cascade (Soykan et al. 2017; Oevel et al. 2024). Intriguingly, depletion of wild‐type htt in cultured neurons or via genomic ablation in cortex results in hyperactivation of Rac1 (Tousley et al. 2019; Wennagel et al. 2022), suggesting loss of wild‐type htt function may release repression of Rac1 resulting in excess ADBE.

How might the observed excess recruitment of ADBE contribute to HD? Typically, reduced endocytosis results in a depression of neurotransmission during high activity (Shupliakov et al. 1997; Chen et al. 2003; Koh et al. 2004; Koo et al. 2015); however, extrapolating the consequences of altered ADBE is more complex, since (1) it does not occur at every synapse (Nicholson‐Fish et al. 2016; Clayton and Cousin 2009), and (2) is only dominant during periods of high activity (Clayton et al. 2008). One potential explanation could arise from emerging molecular links between ADBE and asynchronous release (Raingo et al. 2012; Li et al. 2017; Nicholson‐Fish et al. 2015; Evstratova et al. 2014; Bacaj et al. 2013; Lin et al. 2020). Intriguingly, high‐frequency stimulation of the striatum results in a higher prevalence of this neurotransmitter release mode in two independent HD models (Dvorzhak et al. 2013). However, excess recruitment of ADBE appears to be a universal defect across cell types, so how can this explain selective degeneration of MSNs in HD? It is plausible that the excess recruitment of ADBE alone has no detrimental impact on circuit function. However, when it is coupled with the selective activity‐dependent and striatal‐specific defect in SV cargo trafficking (McAdam et al. 2020), it may comprise a synergistic, “double hit” rendering MSNs neurons exquisitely vulnerable to high activity input in HD.

Finally, CAG expansion on the *HTT* gene also has a role in the manifestation of a developmental phenotype in HD (Humbert 2010). Importantly, compensatory correction of a series of developmental synaptic defects in heterozygous HD mice is reliant on wild‐type htt (Braz et al. 2022). Therefore, the defects in ADBE observed due to loss of htt function may result in altered circuit and brain function in early life. In support, mutations in a series of SV endocytosis genes are causal in neurodevelopmental disorders such as epilepsy, autism and intellectual disability (Bonnycastle et al. 2021). Furthermore, a number of rodent models of both monogenic autism spectrum disorders and epilepsy display defective SV endocytosis (Bonnycastle et al. 2023; Kontaxi et al. 2023) and in some cases dysfunctional ADBE (Bonnycastle et al. 2022, 2025). Therefore, SV endocytosis may be a convergence point in these neurodevelopmental conditions that may provide a route to navigate the developmental consequences of mhtt in early, and potentially later life.

## Author Contributions

Conceptualization: Karen J. Smillie, Michael A. Cousin. Funding acquisition: Karen J. Smillie, Michael A. Cousin. Investigation: Han C.G. Tan, Andrew Morton, Robyn L. McAdam, Karen J. Smillie, Michael A. Cousin. Data analysis: Han C.G. Tan, Andrew Morton, Robyn L. McAdam, Karen J. Smillie, Michael A. Cousin. Methodology: Han C.G. Tan, Andrew Morton, Robyn L. McAdam, Karen J. Smillie, Michael A. Cousin. Resources: Karen J. Smillie, Michael A. Cousin. Supervision: Karen J. Smillie, Michael A. Cousin. Writing – original draft: Karen J. Smillie, Michael A. Cousin. Writing – review and editing: Han C.G. Tan, Andrew Morton, Robyn L. McAdam, Karen J. Smillie, Michael A. Cousin.

## Conflicts of Interest

Michael A. Cousin is a handling editor for the Journal of Neurochemistry.

## Peer Review

The peer review history for this article is available at https://www.webofscience.com/api/gateway/wos/peer‐review/10.1111/jnc.70134.

## Data Availability

The data that support the findings of this study are available from the corresponding author upon reasonable request.
